# Two antimicrobial genes from *Aegilops tauschii* Cosson identified by the *Bacillus subtilis* expression system

**DOI:** 10.1038/s41598-020-70314-5

**Published:** 2020-08-07

**Authors:** Tingting Fu, Md. Samiul Islam, Mohsin Ali, Jia Wu, Wubei Dong

**Affiliations:** Department of Plant Pathology, College of Plant Science and Technology and the Key Lab of Crop Disease Monitoring and Safety Control in Hubei Province, Huazhong Agricultural University, Wuhan, 430070 Hubei Province China

**Keywords:** Drug discovery, Microbiology, Plant sciences

## Abstract

Antimicrobial genes play an important role as a primary defense mechanism in all multicellular organisms. We chose *Bacillus subtilis* as a target pathogen indicator and transferred the *Aegilops tauschii* Cosson cDNA library into *B. subtilis* cells. Expression of the candidate antimicrobial gene can inhibit *B. subtilis* cell growth. Using this strategy, we screened six genes that have an internal effect on the indicator bacteria. Then, the secreted proteins were extracted and tested; two genes, *AtR100* and *AtR472*, were found to have strong external antimicrobial activities with broad-spectrum resistance against *Xanthomonas oryzae* pv. oryzicola, *Clavibacter fangii*, and *Botrytis cinerea*. Additionally, thermal stability tests indicated that the antimicrobial activities of both proteins were thermostable. Furthermore, these two proteins exhibited no significant hemolytic activities. To test the feasibility of application at the industrial level, liquid fermentation and spray drying of these two proteins were conducted. Powder dilutions were shown to have significant inhibitory effects on *B. cinerea*. Fluorescence microscopy and flow cytometry results showed that the purified protein impaired and targeted the cell membranes. This study revealed that these two antimicrobial peptides could potentially be used for replacing antibiotics, which would provide the chance to reduce the emergence of drug resistance.

## Introduction

Antibiotics have been widely used worldwide in recent decades to control many pathogens. Due to the excessive use of antibiotics, many bacteria have already developed antibiotic resistance mechanisms^1,2^. Meanwhile, pathogen resistance has become a serious global threat, especially in intensive care units, where resistance often occurs^3,4^. The increasing number of multi-drug-resistant pathogens has led to a growing demand for new antibiotics; however, even if new antibiotics are identified, resistance cannot be avoided because of antibiotic overuse, which is the primary cause of antibiotic resistance^5,6^. With the increasing use of antibiotics, there is an urgent need to produce something with a negative impact against resistance mechanisms; thus, new therapeutic agents to replace antibiotics must be found. Antimicrobial peptides (AMPs) are potential substitutes for antibiotics because of their broad-spectrum resistance and rare resistant variants^7^. These peptides have broad activity against a variety of bacteria, fungi, viruses, parasites, and cancer cells; furthermore, almost all organisms have a variety of broad-spectrum antimicrobial peptides^8^. An antimicrobial peptide is an amphiphilic, cationic, and small protein in organisms as well as an important component of the innate immune system^9^. The natural immunity of many organisms relies on the invasive power of antimicrobial peptides against different microbes^10^. Since some antimicrobial peptides are biologically safe and are not susceptible to drug resistance, more and more of these have been discovered and excavated^11–14^, and the discovery and application of antimicrobial peptides will continue to increase in the future.

There are many ways to screen antimicrobial genes, proteins, or other compounds; however, some screening methods are used to identify known genes, such as PCR amplification and DNA sequencing^15^, microarray and functional-based screening^16^, fluorescent high-throughput screening^17^, and genome-wide high-throughput screening^18^. These technologies have some beneficial effects as well as some limitations, like high costs, and the advantages of developing new resistance genes are not obvious. Therefore, we need an efficient and low-cost screening method. Recently, by using the *B. subtilis* expression system, Kong et al. developed an efficient method to screen novel antimicrobial proteins^19^. *Escherichia coli* and *B. subtilis* are two kinds of bacteria that have been commonly used in host-vector expression systems in recent years^20,21^. The *E. coli* expression system has become one of the most effective and widely used strategies for recombinant protein production due to its simple genetic operation, low cost, and fast growth^22^, while the *B. subtilis* expression system has received extensive attention for its biosafety, clear genetic background, and strong ability to secrete proteins^23,24^. Both systems can be used to screen for antimicrobial peptides or antimicrobial genes, but our previous research indicates that the expression system of *B. subtilis* is more efficient than that of *E. coli*^19^.

There are many disease resistance genes in plants, which have an inhibitory effect on pathogenic bacteria and exhibit distinctive functions in host–pathogen interactions^19,25^. Here we choose *Aegilops tauschii* (an annual herbaceous plant of the family Gramineae) as the plant material, which is resistant to drought and easily adaptable to the environment^26^. As a material for wheat genetic breeding, *Ae. tauschii* can be crossed with wheat to obtain pathogen-resistant varieties^27^. The resistance genes in *Ae. tauschii* can be retained in the new wheat varieties during the hybridization process to make use of these genes^28,29^.

Our strategy aimed to transform the *Ae. tauschii* cDNA library into the *B. subtilis* expression system and then screen out the antimicrobial gene. We used the pBE-S vector purchased from TaKaRa Co. The pBE-S vector contains a subtilisin promoter (*aprE* promoter) and a secretion signal peptide (*aprE* SP). The secretion signal peptide is derived from *B. subtilis* and is located upstream of the multi-cloning site (MCS). The His-tag sequence is located downstream of MCS^30^, which enables screening of effective secretory signal peptides for target proteins. The screening principle is that the expression of the candidate resistance gene from *Ae. tauschii* in a *B. subtilis* cell can cause autolysis in the cell, and the candidate antimicrobial genes are obtained by observing the autolysis of the *B. subtilis* cells. Here, *B. subtilis* cells are used as a pathogen indicator for resistance gene screening. For functional verification, these antimicrobial candidate genes were expressed again in *B. subtilis* to produce AMPs that are secreted extracellularly; then, the extracted exogenous proteins and various pathogens confront each other, and a broad-spectrum antimicrobial gene can be selected. This technique has been demonstrated again as convenient for isolating antimicrobial genes against not only bacteria, but also fungi^19^.

## Results

### Screening of antimicrobial genes from *Ae. tauschii* using the *B. subtilis* expression system

**Figure 1:**
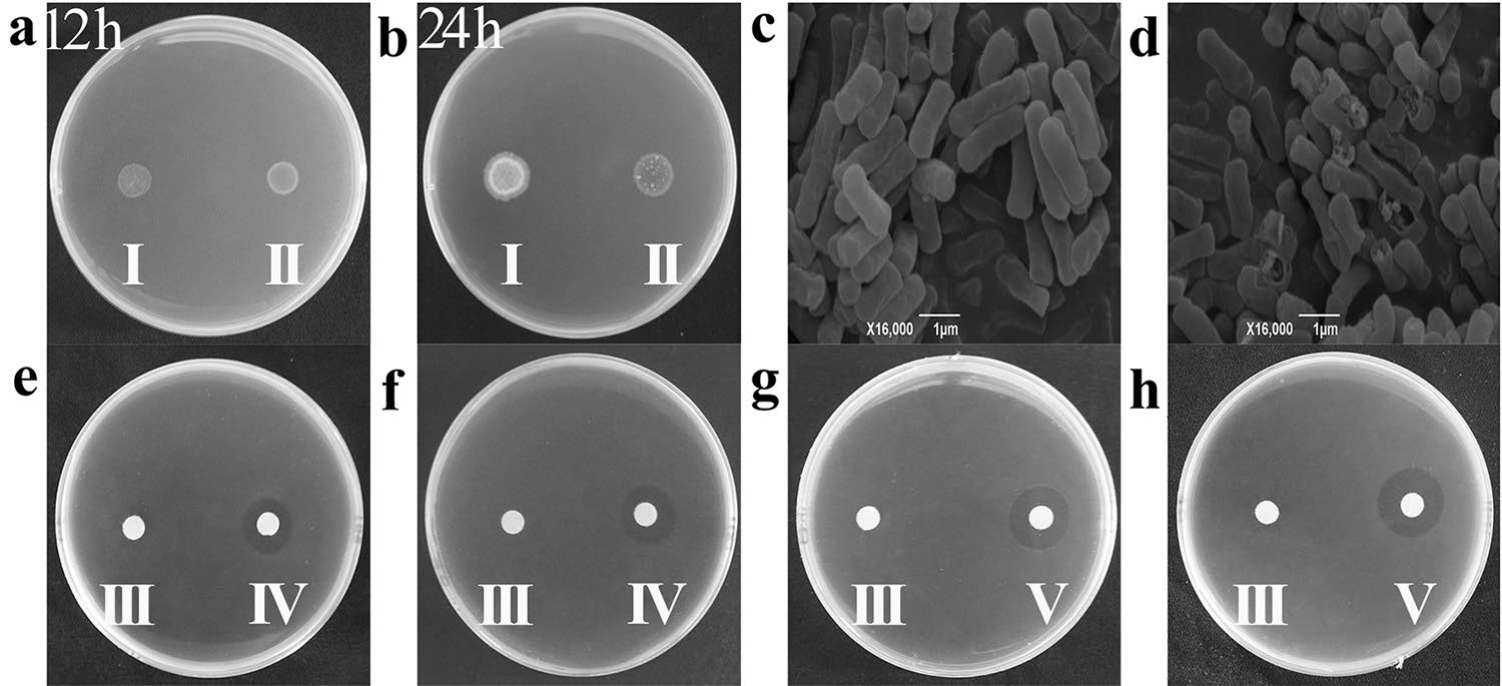
Effects of antimicrobial genes on host cells and the inhibitory activities against different bacterial species. (**a**,**b**) *B. subtilis* strains (harboring an empty vector and *AtR472*, respectively) were separately dropped onto LB plates containing kanamycin. Changes in the non-autolyzed strain (harboring an empty vector) and the autolyzed strain (harboring *AtR472*) were observed on the same plate at 12 h and 24 h, respectively. There were no significant changes in either strain at 12 h (**a**). The non-autolyzed empty vector strain was the control (**I**), and the autolyzed strain harbored *AtR472* (**II**). After 24 h, the *AtR472* strain was autolyzed, whereas the control was not autolyzed (**b**). (**c**,**d**) *B. subtilis* strains (harboring an empty vector and *AtR472*, respectively) under scanning electron microscopy. The empty vector (**c**) caused no change in the host cells; the autolyzed strain *AtR472* secreted the protein and caused host cell damage (**d**). (**e–h**) The antibacterial effects of AtR100 and AtR472 proteins on different bacteria. First, 20 µL of each protein extracted by ammonium sulfate was dropped on the filter paper on each plate, and the inhibition zone was observed within 6–12 h. Indicator bacteria are *B. subtilis* 168 (**e**, **h**), *Clavibater michiganensis* (Smith) (**f**), and *Xanthomonas oryzae* pv*.* oryzicola (**g**). AtR100 (**IV**) and AtR472 (**V**) were the tested proteins, and SCK6 (**III**) was the control. All experiments were repeated three times under normal conditions, and similar results were obtained.

To screen antimicrobial genes, we constructed the *Ae. tauschii* cDNA library using the pBE-S expression vector and transferred it to the *B. subtilis* expression system. Our selection was based on the case that the protein encoded by the antimicrobial gene in the cDNA library has an antimicrobial effect on the host cell and causes it to be damaged (Fig. 1a,b). Here, we obtained six autolyzed clones with good antibacterial effects from more than 1,600 genes. Secreted proteins of these six clones were extracted with ammonium sulfate and screened again with protein for antibacterial experiments, and the two clones *AtR100* (63 bp) and *AtR472* (135 bp) with the most obvious bacteriostatic effects were selected, which showed broad-spectrum resistance to bacteria. The NCBI BLAST analyses of these two clones revealed that they were segments of the *Ae. tauschii* genes, but the peptide sequences were not reported yet (Table S1). *B. subtilis* strains harboring an empty vector (control) or the *AtR472* gene were selected and visualized by SEM after culturing for 36 h (Fig. 1c,d). The empty vector was used as a control and was transferred to *B. subtilis* without any insertion gene. From the above results, it was demonstrated that transferring the pBE-S expression vector into the *B. subtilis* expression system was an efficient method for antimicrobial gene screening.

### Identification of antimicrobial genes for broad-spectrum pathogen resistances

After screening the resistant genes, two proteins, AtR100 and AtR472 were finally screened by the *B. subtilis* expression system, which has better and more stable antibacterial effects. Compared with the control (SCK6), AtR100 and AtR472 showed effective antibacterial assays against Gram-positive and Gram-negative bacteria (Table 1, Fig. 1e–h). Both *AtR100* and *AtR472* showed relatively strong bacteriostatic activity and thus served as candidate genes for the next experiments.

**Table 1:** Antibacterial spectrum of the *Ae. tauschii* antimicrobial genes.

Gene ID	Gram-positive bacteria							Gram-negative bacteria			
	*B. subtilis* IA274	*Clavibater michiganensis*	*B. cereus* 905	*Clavibacter fangii*	*B. subtilis* 330-2	*B. subtilis* RIK1285	*B. subtilis* 168	*Xanthomonas oryzae* pv. oryzae	*X. oryzae* pv. oryzicola	*Rastonia solanacearum*	*X. campestris* pv. holcicola
*AtR100*	−	+++	−	+++	+	−	+++	−	+++	+	−
*AtR472*	−	−	−	+++	+	−	+++	+	+++	+	−

−, Representative effect is not significant; +, Representative effect is significant; +*p* < 0.05, ++*p* < 0.01, +++*p* < 0.001. The results are the mean values from three independent experiments. Significance analysis was performed by SPSS22.0 (SPSS Inc., Chicago, IL, USA).

### Hemolysis analysis of porcine erythrocytes

**Figure 2:**
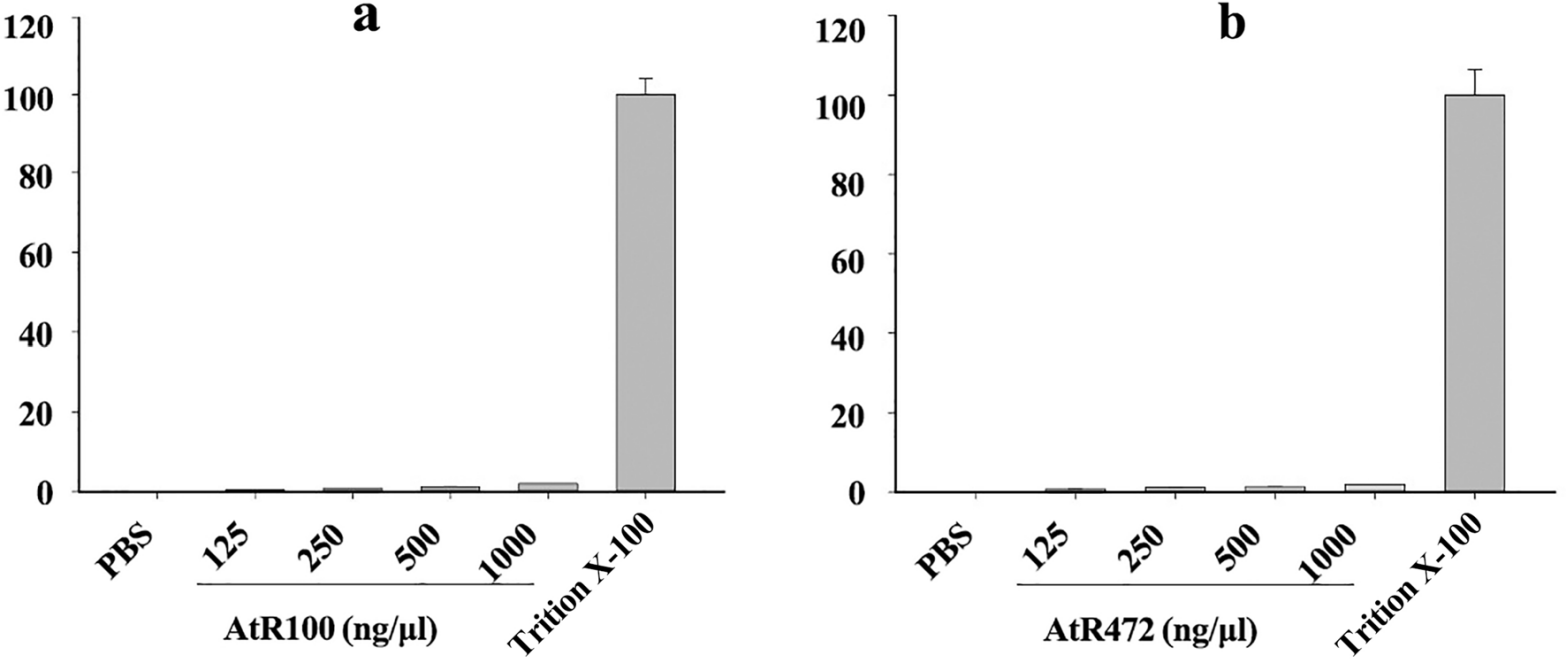
Hemolysis assay of AtR100 and AtR472. Hemolysis of porcine erythrocytes with different concentrations of AtR100 (**a**) and AtR472 (**b**). PBS was used as a negative control; Triton X-100 was used as a positive control. Both AtR100 and AtR472 at different concentrations had no significant hemolytic activity on porcine erythrocytes. The Y-axis represents the percentage of hemolysis in porcine erythrocytes. The experiments were repeated three times.

Red blood cell hemolytic activity tests were performed with both AtR100 and AtR472 proteins. Phosphate-buffered saline (PBS) was used as a negative control (0%), and 0.1% Triton X-100 was used as a positive control (100%). The protein concentration range was 125–1,000 µg/mL. Both proteins showed no significant hemolytic activity on porcine erythrocytes even at the highest concentration of 1,000 µg/mL (Fig. 2); at this concentration, the percentages of hemolysis with AtR100 and AtR472 were 2.09% and 1.97%, respectively (Table S2). Therefore, we believe that these two proteins are biologically safe and may be used to develop drugs.

### Determination of thermal stability of antimicrobial proteins

**Figure 3:**
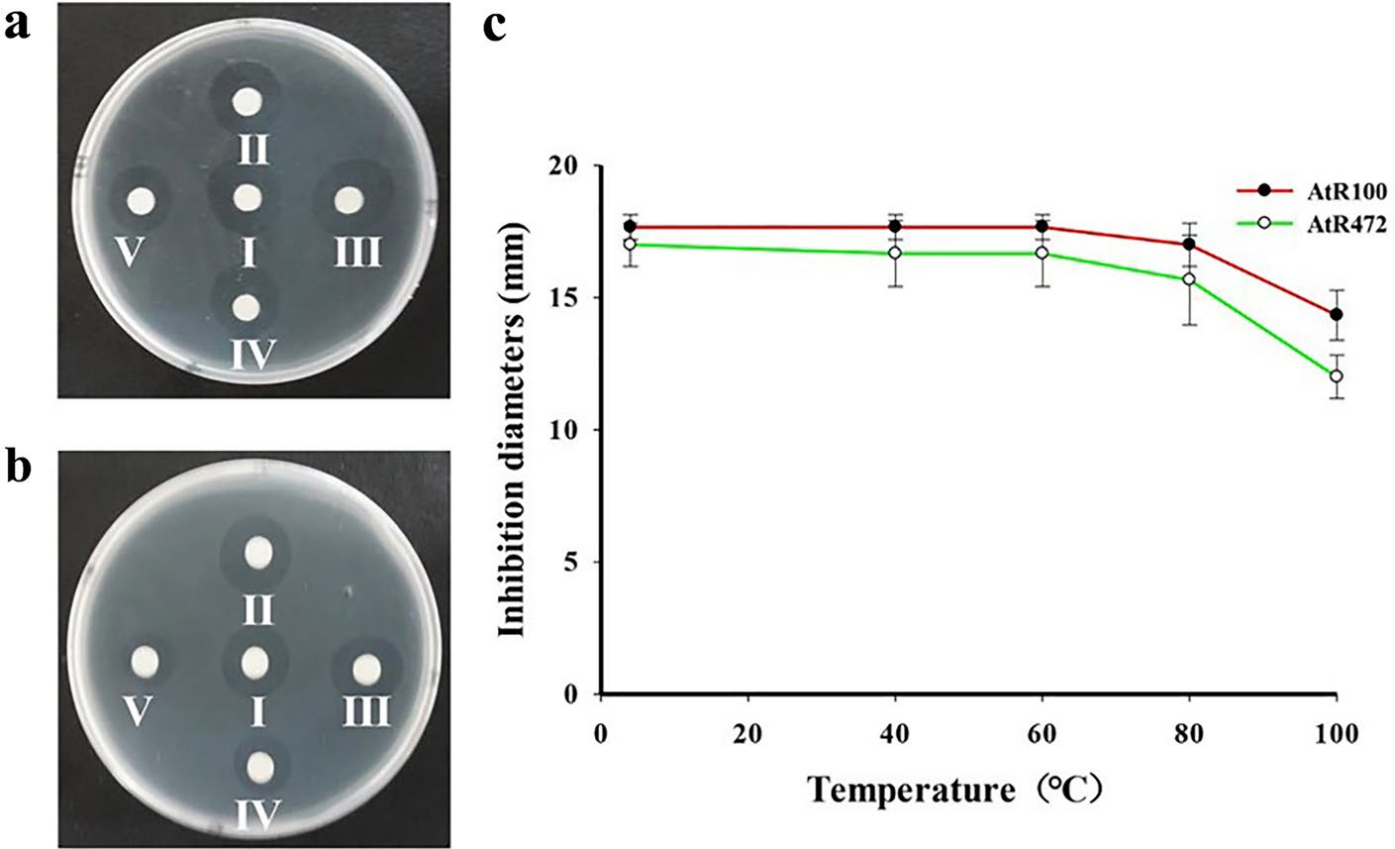
Thermal stability of AtR100 and AtR472. AtR100 (**a**) and AtR472 (**b**) were confronted with *B. subtilis* 168 after heating at 4 °C (**I**), 40 °C (**II**), 60 °C (**III**), 80 °C (**IV**), and 100 °C (**V**). (**c**) The curve of the inhibitory diameter of the two proteins, AtR100 and AtR472, against the rising temperature. The results showed that both proteins were thermostable to some extent. Experiments were repeated three times.

The two proteins AtR100 and AtR472 were treated at 4, 40, 60, 80, and 100 °C for 15 min, and they showed stable antibacterial activities at all high temperatures (Fig. 3). These proteins were as thermally stable as some other antimicrobial peptides; thus, they have the potential to be used as antibacterial drugs.

### Evaluation of the engineered *B. subtilis* liquid fermentation products

After verifying the biological activity of these two antibacterial peptides, we attempted to test their mass production and usage by performing a large-volume liquid fermentation. The *B. subtilis* strains harboring an empty vector, *AtR100*, and *AtR472* were separately cultured by liquid fermentation to obtain fermentation broths, which were made into powder products by spray drying. To effectively evaluate the titer of the engineered *B. subtilis* powder, an appropriate series of dilutions of the powder was prepared with sterile water; generally, three dilutions were used. Then, 100 µL of each dilution was spread on solid LB media and placed in a 37 °C incubator for 1 d. After incubation, the colony forming units (CFU) were counted as 6 × 10^9^ CFU/g (empty vector), 1 × 10^10^ CFU/g (*AtR100*), and 4 × 10^9^ CFU/g (*AtR472*).

### Antifungal activities of the AtR100 and AtR472 powder

**Figure 4:**
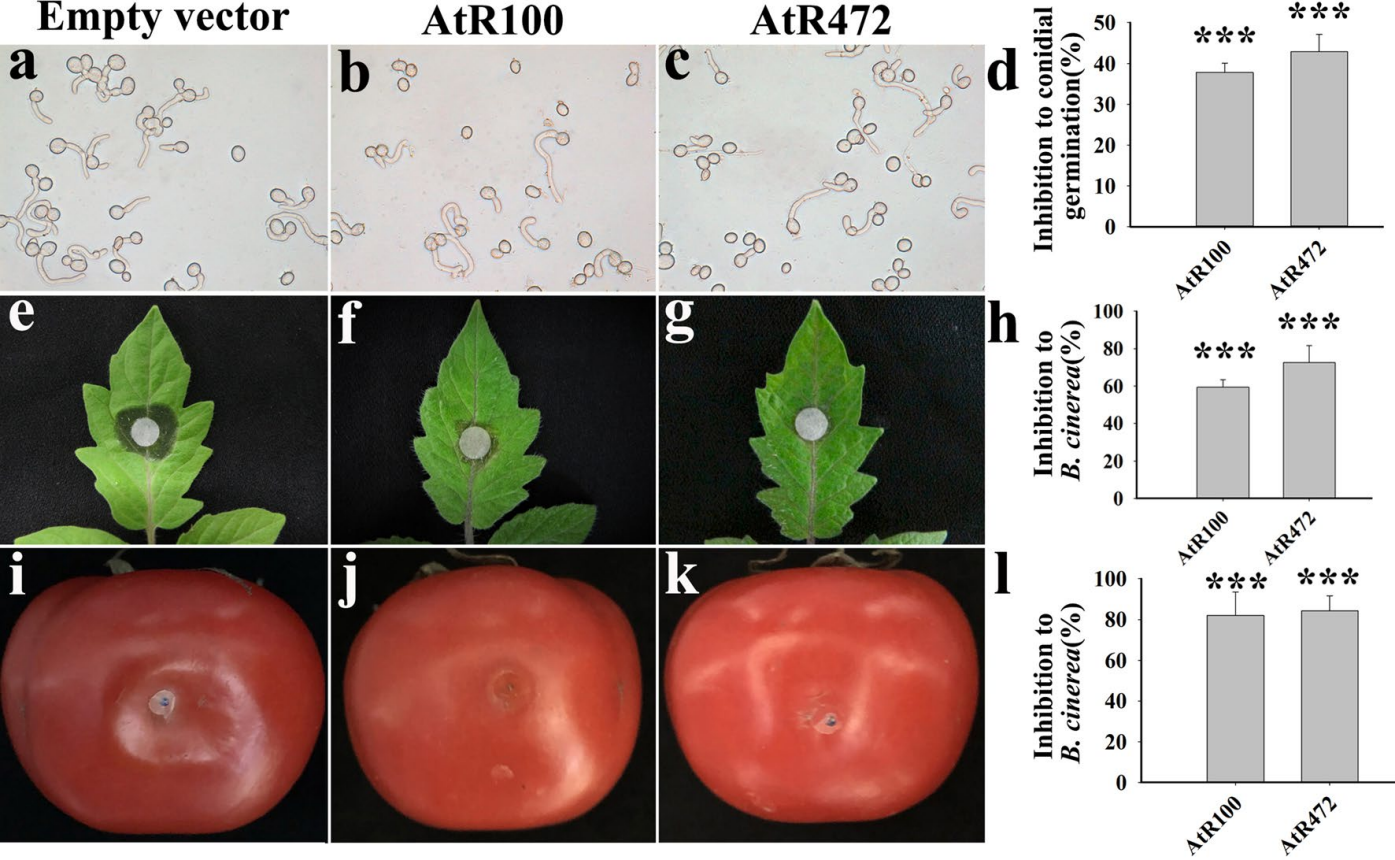
Activities of AtR100 and AtR472 against *B. cinerea*. (**a-d**) Effects of AtR100 and AtR472 on the germination of *B. cinerea* conidia. Conidial germination after treatment (5 h) with empty vector (**a**), AtR100 (**b**), and AtR472 (**c**). (**d**) Inhibition rates of AtR100 and AtR472 toward *B. cinerea* conidial germination. (**e–h**) AtR100 and AtR472 treatments inhibited conidia infection after inoculation on tomato leaves. The infection of tomato leaves after treatment with empty vector (**e**), AtR100 (**f**), and AtR472 (**g**) for 3 d, respectively. (**h**) The inhibition rates by AtR100 and AtR472 treatments after inoculating conidia on tomato leaves. (**i–l**) AtR100 and AtR472 treatments inhibited the *B. cinerea* mycelium inoculated on tomatoes. The decay of tomatoes treated with empty vector (**i**), AtR100 (**j**), and AtR472 (**k**) for 3 d. (**l**) The inhibition rates by AtR100 and AtR472 treatment after inoculating tomatoes with mycelium. Data are representative of repeated experiments, and significance analysis was performed using one-way analysis of variance and Duncan’s multiple comparison tests by SPSS22.0, ***p < 0.001. All experiments were repeated three times.

Each fermented *B. subtilis* powder harboring *AtR100*, *AtR472*, or empty vector was diluted to 1 × 10^6^ CFU/mL, and the *B. subtilis* cells in the diluted solution exerted an antimicrobial action by secreting antimicrobial proteins. The conidial suspension of *B. cinerea* was mixed with diluted *B. subtilis* powder (1:1 ratio), and conidial germination was observed after 5 h. The results showed that most conidia treated with the control (empty vector) germinated; however, only a few conidia treated with AtR100 or AtR472 showed germination (Fig. 4a–c). AtR100 and AtR472 have high inhibition rates toward conidial germination (Fig. 4d). The mixtures of the conidial suspension and each of the three *B. subtilis* strains were inoculated separately onto filter paper on tomato leaves, and infection of the leaves was observed after 3 d. The results indicated that the leaves treated with the control (empty vector) were severely affected, whereas those treated with AtR100 or AtR472 showed mild infection (Fig. 4e–g). The inhibition rates toward *B. cinerea* showed that both AtR100 and AtR472 proteins had good inhibitory effects on tomato leaves (Fig. 4h). The *B. cinerea* mycelium was inoculated onto tomato fruit, and 20 μL of diluted engineered *B. subtilis* powder was sprayed on *B. cinerea*. The degree of tomato rot was observed in the affected area after 3 d. The results indicated that the control (empty vector) area was severely rotted, while the AtR100 and AtR472 treatments caused less rot (Fig. 4i–k). The inhibition rates toward the *B. cinerea* mycelium on tomatoes also revealed that AtR100 and AtR472 had a significant inhibitory effect on *B. cinerea* (Fig. 4l).

### Bioinformatics analysis of antimicrobial proteins

Bioinformatics analysis can aid in determining the relevance between protein function and structure. Translational analysis revealed that protein structure is closely related to the function of antimicrobial peptides. The number of amino acids in these six antimicrobial peptides varied from 13 to 45; additionally, their complex structures could be predicted using secondary structure prediction tools^31^. Using these tools, we found that AtR100 and AtR472 have both α-helices and β-strands, whereas the other four proteins contain only β-strands (Table 2). According to our antibacterial results, proteins with both α-helix and β-strand structures were more effective than those with only β-strands. Therefore, we speculate that the α-helix structure may be related to antibacterial activity. According to predictions, most of these proteins are amphoteric or hydrophobic. Among all six proteins, three contained disulfide bonds (Table 2).

**Table 2:** Bioinformatics prediction of antimicrobial peptides.

Protein name	AA no	S–S no	Secondary structure	MV	pI	GRAVY
AtR31	15	0	β-strand	1861.19	8.60	0.127
AtR78	20	1	β-strand	2,282.63	6.89	0.270
AtR222	13	0	β-strand	1595.96	6.73	1.577
AtR352	16	0	β-strand	1917.31	8.75	0.719
AtR100	21	1	α-helix β-strand	2,432.75	5.97	0.148
AtR472	45	2	α-helix β-strand	5,210.08	8.42	0.158

AA No., number of amino acids; GRAVY, grand average of hydropathicity; pI, theoretical pI; MV, molecular weight; S–S No., number of cysteine disulfides.

### Effect of purified AtR100 protein on cell membranes

**Figure 5:**
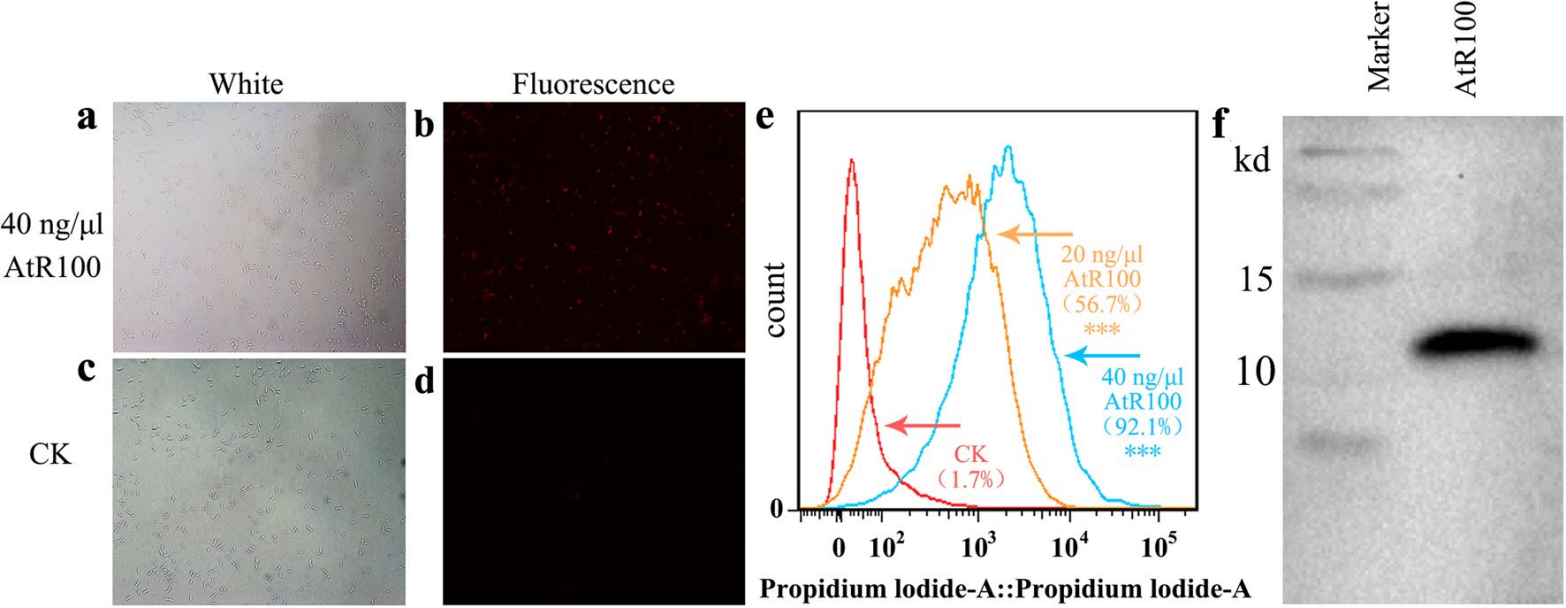
PI uptake and western blot. (**a–d**) The result of PI staining under a fluorescence microscope. *B. subtilis* 168 was incubated with purified AtR100 protein (**a**, **b**) and PBS (**c**,**d**) at 37 °C with shaking (150 rpm/min, 1.5 h). (**a**,**c**) *B. subtilis* 168 under white light. (**b**,**d**) *B. subtilis* 168 under fluorescence. (**e**) PI staining results of flow cytometry after incubating *B. subtilis* 168 with PBS, 20 ng/μL AtR100, and 40 ng/μL AtR100. (**f**) Western blot of purified AtR100 protein expression. The full-length blot was presented in Supplementary Fig. S3. Data are representative of repeated experiments, and significance analysis was performed using one-way analysis of variance and Duncan’s multiple comparison tests by SPSS22.0, ***p < 0.001. Experiments were repeated three times under the same conditions.

We obtained the AtR100 protein via His-tag purification. The purified protein was used to further investigate the effect on the cell membrane. Propidium iodide (PI) can enter the cell through a damaged cell membrane and appears red under fluorescence. The fluorescence microscopy results indicated that nearly all bacteria treated with AtR100 protein were stained with PI and exhibited red fluorescence, whereas those treated with the negative control (PBS) showed no fluorescence (Fig. 5a–d). The proportion of fluorescence in cells after treatment with AtR100 protein and PBS was determined by flow cytometry (Fig. 5e). After treatment with the control (PBS), 20 ng/μL AtR100, and 40 ng/μL AtR100, fluorescence was observed in 1.7%, 56.7%, and 92.1% of *B. subtilis* cells, respectively. The results revealed that the purified protein had a destructive effect on the cell membrane integrity of *B. subtilis* 168, and cell membrane damage increased as the protein concentration increased. We also performed western blot analysis on the purified protein and found that its molecular weight was about 11 kD (Fig. 5f).

## Discussion

There are many antimicrobial peptides in nature. These peptides play a key role in the biological innate immune system and help the organism to resist the invasion of pathogenic bacteria; in spite of this, few antimicrobial peptides are used in clinical treatment^32^. Polypeptides are produced by ribosomes, such as ribosomally synthesized and post-translationally modified peptides (RiPPs), or by non-ribosomal peptide synthetases (NRPS). Polypeptides produced by NRPS are generally resistant to proteases, and RiPPs are natural products with diverse structures and biological activities^33,34^. Here, we obtained ribosomally synthesized peptides. In fact, antimicrobial peptides can be used as a substitute for some drugs to avoid drug resistance. Although there are many antimicrobial peptides in nature, they are quite challenging to isolate and identify. In this study, the *B. subtilis* expression system was used to screen for antimicrobial genes from *Ae. tauschii*; we chose this system for antimicrobial peptide screening due to its numerous advantages. First, the cell-free transcription-translation system makes *B. subtilis* an important model organism^35^. Secondly, *B. subtilis* can be a useful expression vector for antimicrobial peptides through inserting specific genes to secrete specific proteins^36^. In addition, the yield of heterologous proteins is very important in research and practical applications^37^; therefore, it is necessary to choose a system with a high expression capacity. Expression systems such as *E. coli*, *B. subtilis*, *Saccharomyces cerevisiae*, and mammalian cells are often used for various production applications^38–41^. Compared with other expression systems, *B. subtilis* has the advantages of non-toxicity, strong secretion ability, and easy fermentation and culturing^42^. In previous studies, *B. subtilis* produced antimicrobial peptides, such as surfactins, iturins, and fengycins, and antibacterial peptides could be obtained by an ammonium sulfate method^43,44^; we also used this method for crude protein extraction in some experiments, but we strictly used SCK6 as a control. In our experiments, an overabundance of SCK6 crude protein resulted in an inhibition zone, indicating antimicrobial function. For each test pathogen strain, we adjusted the concentration of SCK6 crude protein to minimize its antimicrobial effect, and we strictly used the same protein quantities with test treatments. *B. subtilis* SCK6 is a highly efficient protein expression strain^45^. We chose *B. subtilis* SCK6 as the expression host strain because the results of our preliminary experiments showed that its transformation efficiency was higher than that of *B. subtilis* WB800, and the genes we screened via the *B. subtilis* SCK6 strain were verified for protein production by transforming them into the *B. subtilis* WB800 strain.

The results shown in Table 1 and Fig. 1e–h demonstrate that the proteins have good antibacterial activities. Pathogenic bacteria cause serious economic losses in production. Excessive use of chemical pesticides results in damage to the ecological environment and easily causes resistance in pathogenic bacteria. Moreover, chemical control leads to hazardous effects on human health through the food chain. If biological control is used to replace a part of chemical control, it will be greatly advantageous for both the ecological environment and pathogen control. Studies have shown that antimicrobial peptides inhibit many pathogens^13^; if these peptides are applied to crop production, they could be used as a substitute for chemical pesticides to control pathogenic bacteria.

If an antimicrobial peptide is intended to serve as an alternative to antibiotics or pesticides, further development and applications are needed, and its safety and stability must be tested. Accordingly, the two antimicrobial peptides screened by the B. subtilis expression system showed no significant hemolytic activity on porcine red blood cells (Fig. 2), which indicates that these two peptides effectively inhibit bacteria but are safe for other organisms. We also tested the thermal stability of the antimicrobial peptides and found that they retained significant bacteriostatic activity after 15 min of treatment at various temperatures (Fig. 3). This indicates that the two antimicrobial peptides can adapt to different temperatures in the natural environment and thus are valuable for further development and application.

The function of a protein is closely related to its secondary structure. Knowing the secondary structure of an antimicrobial peptide is useful for understanding its biological activity; for example, an antimicrobial peptide with an α-helix structure is generally considered to have a potential function toward cell membranes^46^. We predicted the biological information of the six screened peptides; combined with our antibacterial results, it was found that proteins with both α-helix and β-strand structures have more significant antibacterial effects than those with only β-strand structures (Table 2). This was consistent with the fact that most antimicrobial peptides contain an α-helix structure. Western blot results showed that the molecular weight of AtR100 was about 11 kD (Fig. 5f), which was larger than the 2.4 kD predicted by bioinformatics analysis. It is possible that the vector and His-tag increase the molecular mass of the protein. Previous studies have shown that the molecular weight of a protein labeled with His-tag will be greater than the predicted weight^47^, and the discrepancy has also been discussed by Kong et al.^19^.

Most antimicrobial peptides act directly on the cell membrane, which is how the mechanism of action differs between antimicrobial peptides and antibiotics. To confirm whether the antimicrobial peptide inhibits bacteria by destroying the cell membrane of *B. subtilis* 168, we observed changes using fluorescence microscopy and PI staining after incubating the cells with purified proteins. No difference was found after treatment with purified AtR100 and PBS under white light (Fig. 5a,c), but *B. subtilis* 168 treated with purified AtR100 showed red fluorescence (Fig. 5b), whereas the control (PBS) did not (Fig. 5d). The results showed that the cell membrane was destroyed in most *B. subtilis 168* treated with the protein. Flow cytometry also demonstrated that most *B. subtilis* 168 incubated with the purified AtR100 showed fluorescence; as the purified AtR100 concentration increased, the proportion of fluorescence also rose (Fig. 5e). The direct destruction of bacterial cell membranes by antimicrobial peptides confirms that antimicrobial peptides can be an alternative approach to antibiotics. Antibiotics generally act on a target, and the ribosome is the major target for most antibiotics^48^, such as tetracycline, pactamycin, and hygromycin B. These antibiotics mainly target the ribosomal 30S subunit^49^. Compared to antibiotics with specific targets, antimicrobial peptides directly damage cell membranes and cause damage to bacteria, making the development of resistance less likely. Therefore, we believe that antimicrobial peptides can be used as an alternative to antibiotics in future drug development.

If an antimicrobial peptide is applied, it will be used as a biological control agent to control pathogens. Biological control has the advantages of environmental and biological safety, which cannot be achieved by chemical control. People urgently need to understand biological control and avoid excessive dependence and the use of chemical control to decrease resistance. At present, the use of bacteria as a biological control agent is slowly being promoted for production, and *B. subtilis* is one of them. In addition to inhibiting bacteria, *B. subtilis* also has antifungal activities^50^, and in recent decades, antimicrobial substances produced by *B. subtilis* have also been used in food processing and crop protection^51^. Since antimicrobial peptides exhibit good antibacterial effects and are safe, we have tried to apply antimicrobial peptides in production. Liquid fermentation can produce *B. subtilis* in large quantities, and it is often used in production^52,53^; thus, we chose to use these technologies to produce our *B. subtilis* strain. The engineered *B. subtilis* powder obtained via spray drying was found to have activity against *B. cinerea*. Mixing the powder with a conidial suspension inhibited conidia germination (Fig. 4a–d). In addition, the conidial suspension of *B. cinerea* mixed with the engineered *B. subtilis* powder inhibited conidial infection on tomato leaves (Fig. 4e–h). The powder also inhibited *B. cinerea* mycelium infection on tomatoes (Fig. 4i–l). The engineered *B. subtilis* strains harboring AtR100 and AtR472 genes were prepared into a powder by liquid fermentation and spray drying, which is more convenient and conducive to the application and preservation of these two pure antimicrobial peptides. Therefore, the antibacterial peptides screened by our *B. subtilis* expression system can be used together with the *B. subtilis* strain, which is convenient for production and application.

In summary, we used the *B. subtilis* expression system to efficiently screen for some antimicrobial peptides. As a pathogen indicator, *B. subtilis* cells can be used to screen for antimicrobial peptides. Although there is a limitation that some strong AMPs can kill *B. subtilis* cells too quickly to detect the desired genes, the *B. subtilis* screening strategy is able to isolate and clone for broad-spectrum antimicrobial peptides^54^. By screening cDNA libraries and observing autolyzed clones, it is feasible to rapidly identify resistance genes. These candidate genes are expressed by *B. subtilis*, which acts as a production vector to obtain the secreted proteins for pathogen prevention. Finally, antimicrobial peptides having broad-spectrum resistance were obtained. In this antimicrobial protein production, one advantage is that the *B. subtilis* is biologically safe.

## Materials and methods

### Planting *Ae. tauschii* and inoculation of pathogens

*Aegilops tauschii* seeds are difficult to germinate during the dormant period; therefore, the seeds were placed in an oven at 45 °C for 8 h. Then the outer shell was peeled off, and the seeds were soaked in water for 2 d and placed in soil after germination. When the plants grew to the seedling stage, inoculation of bacteria was initiated. *Rhizoctonia solani* WH1 was cultured in a 28 °C culture chamber, and the hyphae were placed in PDB. After culturing for 2–3 d at 150 rpm, the hyphae were filtered with gauze, and a measuring cup was used to catch the PDB. The hyphae on the gauze were ground in a mortar, poured into ultrapure water, and stirred with a glass rod. The plant topsoil was pulled out of the white roots, and a small amount of the hyphae dissolved in water was poured twice onto the root surface of the plant. Then, the PDB in the measuring cup was poured on the topsoil to provide nutrition to the hyphae.

### Construction of the cDNA library

*Rhizoctonia solani* was inoculated on *Ae. tauschii* leaves, which were then collected. RNA was extracted from the leaves with Trizol, mRNA was purified from the total RNA, and then cDNA was synthesized. The cDNA was ligated by T_4_ ligase to an adaptor containing *Nde* I cleavage sites. Then, the cDNA containing the *Nde* I cleavage sites and the vector pBE-S were digested with *Xba* I and *Nde* I, after which the cDNA and pBE-S were ligated with T_4_ ligase. The ligation mixture was transformed into competent *E. coli* HS-T08 cells, and the plasmid was extracted. Finally, the plasmid was transformed into competent *B. subtilis* SCK6 cells. Detailed steps can be found in the Supplementary Information. The primers for cDNA library construction can be found in Table S3. Total RNA, mRNA, and cDNA images are shown in Supplementary Fig. S2.

### Quality assessment of the cDNA library

To check the quality of the cDNA library, 96 monoclonal clones were randomly selected from *B. subtilis* for PCR amplification under the following conditions: 95 °C for 5 min, 95 °C for 30 s, 55 °C for 30 s, 72 °C for 1 min (28 cycles), and 72 °C for 5 min. The PCR product was visualized by electrophoresis, and the sequence was analyzed. The quality of the cDNA library is depicted in Supplementary Fig. S1.

### Identification of cDNA libraries

All single colonies were picked up with a toothpick, shaken on LB medium containing kanamycin (180 rpm, 5–8 h), and stored in a − 70 °C refrigerator with glycerol. A bacterial droplet (1 µL) was pipetted from each tube onto an LB plate containing kanamycin, and colony autolysis was observed (Fig. 1a,b). The autolyzed strains were recorded, and changes in autolysis were continuously observed for these strains, which were repeated three or more times to determine the stability of the autolysis phenomenon.

### Scanning electron microscopy

Scanning electron microscopy (SEM) of bacterial surface changes was conducted as described^55^; detailed steps including culturing, fixation, and dehydration can be found in the Supplementary Information.

### Protein extraction from the *B. subtilis* expression system

Proteins secreted by the *B. subtilis* expression system were extracted by ammonium sulfate precipitation^56^. Detailed protein extraction methods can be found in the Supplementary Information.

### Hemolysis analysis of porcine erythrocyte

AtR100 and AtR472 proteins were incubated with porcine red blood cells. PBS was used as a negative control, and Triton X-100 was used as a positive control. The percentage of hemolysis was calculated according to the following equation: percent hemolysis = [(Abs450 nm in the peptide solution − Abs450 nm in PBS)/(Abs450 nm in 0.1% Triton X-100 − Abs450 nm in PBS)] × 100. Detailed steps can be found in the  Supplementary Information.

### Antibacterial bioassay

According to the method of Xiao et al.^57^, filter paper was used to observe the antibacterial effect of the antimicrobial protein. First, the solid NA medium was poured into a 90 mm culture dish, and then 400 μL of the bacterial broth was added to the semi-solid NA (55 °C) and covered on the NA plate. After the semi-solid NA solidified, a piece of 6 mm filter paper was placed in the upper layer using tweezers. Protein droplets (20 µL) were pipetted onto the filter paper. The plates were placed in an incubator according to the culture temperature of the bacteria (5–8 h), and then the diameter of the inhibition zone was measured.

### Liquid fermentation and spray drying

The *B. subtilis* culture solution was fermented in a 10 L fermenter. After fermentation was completed, the liquid fermentation broth was dried into a powder using a spray-drying machine. Detailed liquid fermentation and spray drying methods can be found in the Supplementary Information.

### Antifungal bioassay

The control, AtR100, and AtR472 were adjusted to the same concentration according to the CFU of *B. subtilis* powder diluted with sterile water. The adjusted conidial suspension was mixed 1:1 with the diluted *B. subtilis* powder, and 10 μL of the mixed droplets were pipetted onto a hydrophobic slide at 22 °C for 5 h to observe conidial germination. Filter paper was placed on the tomato leaves, and 20 μL of the mixture of conidial suspension and *B. subtilis* were placed on a filter paper, moisturized, and placed in a 20 °C incubator. Infection of the leaves was observed after 3 d. The tomato epidermis was pierced with a needle, and the *B. cinerea* mycelium was added to the wound. Then, 20 µL of the diluted *B. subtilis* powder was pipetted on the affected area, which was wrapped in plastic film, and the tomato decay was observed after 3 d. The conidial suspension was determined according to the method of Jemric et al.^58^. The detailed steps for preparing the conidial suspension and conidial germination can be found in the Supplementary Information.

### Prediction of candidate protein sequences

Protein sequence translation was conducted by the EMBOSS Programs (https://www.ebi.ac.uk/Tools/emboss/). The secondary structure of the antimicrobial protein was predicted by the PSSpred website (https://zhanglab.ccmb.med.umich.edu/PSSpred/). Disulfide bond prediction was measured using DISULFIND (https://disulfind.dsi.unifi.it/)^59^. Online ExPASy tools were used to predict the pI, molecular mass, and grand average of hydropathicity of the predicted proteins (https://expasy.org/tools). The above sequence prediction websites were based on the method of Kong et al.^19^.

### Protein purification and western blot

The recombinant protein AtR100 was isolated by the Ni–NTA His Bind Resin Kit. A mixture of 20 μL protein and 5 μL 5 × protein loading buffer was heated in boiling water for 10 min. Then, 15 μL of the mixed sample was separated by polyacrylamide gel electrophoresis using a Trisine-SDS-PAGE Kit. The western blot step was performed as described^60^; details on the western blot procedure can be found in the Supplementary Information.

### Fluorescence microscopy and flow cytometry analysis

For confocal microscopy, bacteria were prepared as described by Xie et al.^61^. Bacteria were collected from the bacterial log phase (1 × 10^8^ CFU/mL, 3,200 × g, 10 min) and then washed three times with PBS. After washing, the cells were incubated with 40 ng/μL of purified AtR100 for 1.5 h, and PBS was used as a negative control. Then, 10 µg/mL PI was added, the mixture was incubated in the dark (4 °C, 30 min), and 4 µL was spotted onto a slide. Imaging was conducted by a fluorescence microscope (NIKON ECLIPSE 80i, NIKON, Tokyo, Japan). Flow cytometry was also prepared by the above method, and fluorescence data were measured by the FACSVerse flow cytometer (BD Biosciences, Franklin Lakes, NJ, USA).

### Ethics statement

Porcine blood from Landrace and Large White pig hybrids (8 kg) were obtained from the College of Veterinary Medicine, Huazhong Agricultural University. The animal study was approved by the Scientific Ethics Committee of Huazhong Agricultural University. All experiments were performed in compliance with the International Guiding Principles for Biomedical Research Involving Animals.

## Supplementary information

Supplementary Information.
