# Supplementary material for: Two antimicrobial genes from Aegilops tauschii Cosson identified by the Bacillus subtilis expression system
Source: Sci Rep. 2020 Aug 7;10:13346. doi: 10.1038/s41598-020-70314-5 (PMC7414872; doi:10.1038/s41598-020-70314-5)
Supplement: Supplementary file 1 — Supplementary Information. [file 41598_2020_70314_MOESM1_ESM.pdf]

Supporting Information

Two antimicrobial genes from *Aegilops tauschii* Cosson identified by the *Bacillus subtilis* expression system

Tingting Fu, Md. Samiul Islam, Mohsin Ali, Jia Wu, Wubei Dong

Department of Plant Pathology, College of Plant Science and Technology and the Key Lab of Crop Disease Monitoring & Safety Control in Hubei Province, Huazhong Agricultural University, Wuhan, Hubei Province, 430070, China.

*Corresponding author
E-mail: dwb@mail.hzau.edu.cn

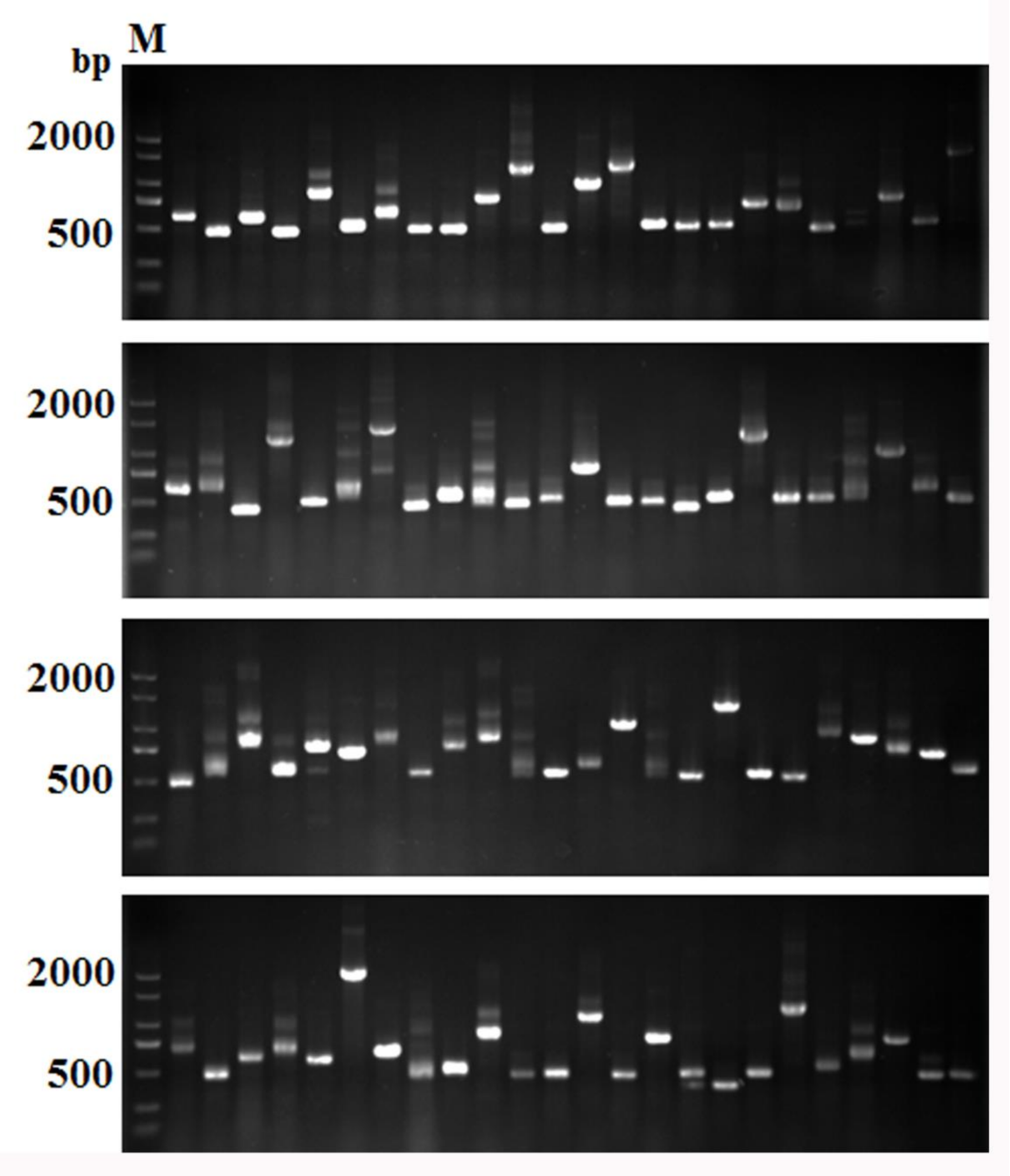

Figure S1. *Aegilops tauschii* cDNA library insert sizes. cDNA library of *B. subtilis* expression system was inserted between 500 - 2000 bp. Marker is the DL2000.

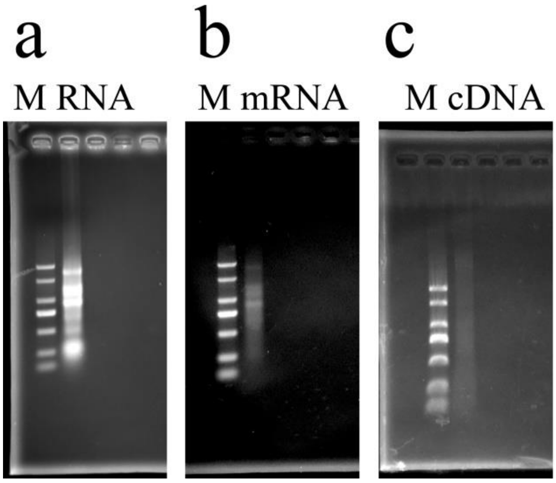

Figure S2. Quality of total RNA, mRNA, and cDNA. The quality of total RNA (a), mRNA (b), cDNA (c) of *Ae. tauschii* cDNA library. Marker of (a-c) is the DL2000.

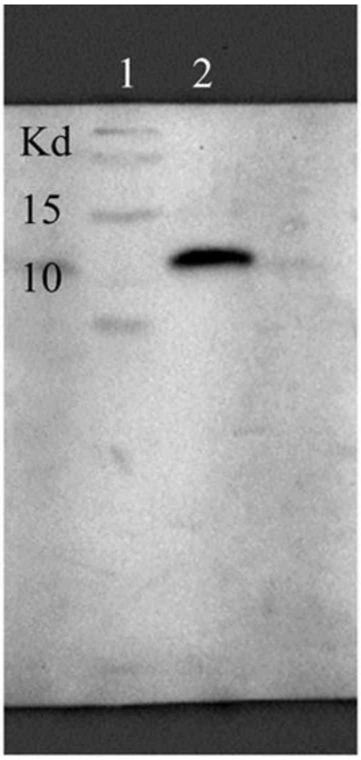

Figure S3. Original full-length western blot of the AtR100 protein. The color pre-staining ultra-low molecular weight (1.7 - 40 Kd) maker showed in lane 1 and the AtR100 protein in lane 2.

Table S1. Amino acid sequences of *Ae. tauschii* antibacterial proteins

Protein ID	Amino acid sequences	Amino acid number
AtR31	RITTLAIHTHIYYMQ	15
AtR78	YHTCTCIYIGLWPHGNTSCL	20
AtR100	QNLCNIYYIQHADHCLAVINS	21
AtR222	LILYYQIHILFCG	13
AtR352	FARSPYNVMMLFISLQ	16
AtR472	LCVSYSVRPSCKFVGRSNWKRNTCLEWCDQLFLFCFLIGGEMANS	45

Table S2. The hemolytic activity of AtR100 and AtR472 proteins on porcine red blood cells

Treatments with	Percentage of hemolysis at different concentrations (µg/ml)			
	125	250	500	1000
AtR100	0.49	0.98	1.35	2.09
AtR472	0.73	1.11	1.35	1.97
PBS	0	0	0	0
Triton X-100	100	100	100	100

Table S3. Primers for *Ae. tauschii* cDNA library construction using the *B. subtilis* expression system

Oligo dT (contains *X*ba I enzyme site)	ACAGGCTCTAGAGCTTTTTTTTTTTTTTTTTTTTTT
Adapter 1 (contains *N*de I Cleavage site)	CTCGAGAGGAATTCCATATGC
Adapter 2 (contains *N*de I Cleavage site)	GCATATGGAATTCCTCTCGAGTACG
Adapter 3 (contains *N*de I Cleavage site)	CTCGAGAGGAATTCCATATGCT
Adapter 4 (contains *N*de I Cleavage site)	AGCATATGGAATTCCTCTCGAGTACG
Adapter 5 (contains *N*de I Cleavage site)	CTCGAGAGGAATTCCATATGCTA
Adapter 6 (contains *N*de I Cleavage site)	TAGCATATGGAATTCCTCTCGAGTACG

Supplementary methods

Construction of *B. subtilis* system cDNA library

The leaves of *Ae. tauschii* were inoculated with *R. solani*, and the leaves were collected every 8 h within 0-4 d. Total RNA was extracted with Trizol reagent, and mRNA was purified using Promega's PolyATtract mRNA Isolation Systems kit. A cDNA library was synthesized using Takara's PrimeScript Double Strand cDNA Synthesis Kit (Oligo dT primer containing *Xba* I cleavage site), and then the cDNA was ligated to *Nde* I adaptor. The cDNA and pBE-S were double-digested with *Xba* I and *Nde* I restriction endonucleases, cDNA was collected using Omega Clean Recovery Kit, and linear pBE-S was used with TRANS GEN Glue Recovery Kit. The cDNA library was ligated with pBE-S using T4 DNA ligase. The ligation mixture was first transferred to *E. coli* HS-T08 for replication, and the growing colony population was subjected to plasmid extraction and then transferred to *B. subtilis* SCK6. Incubate for 12 h in LB plates containing kanamycin (10 μg/ml). Single colonies were picked and shaken in LB containing kanamycin, and glycerol was added to save the strains at a -80 ℃ refrigerator.

SEM

The empty vector and *AtR472* strains stored in the -80 ℃ refrigerator were taken out and streaked on LB plates containing kanamycin, and cultured at 37 ℃ for 12 h. Single colonies were picked and shaken at 180 rpm for 36 h in LB containing kanamycin. The 25 ml of the culture solution were centrifuged in a 50 ml centrifuge tube at 5000 rpm for 6 min. The supernatant was decanted, washed three times with PBS, and finally transferred to a 2 ml EP tube. The supernatant was removed by centrifugation, fixed with 2.5% glutaraldehyde, shaken, and allowed to stand at room temperature for 2 h. Wash with 1 ml of a concentration of 30%, 50%, 70%, and 90% ethanol, shake well, 8000 rpm, 3-5 min centrifugation. Finally, it was dehydrated with 1 ml of 100% ethanol, and dried by a freeze dryer. After coated with gold on the sample, a JSM-7001F (JEOL Japan) Scanning Electron Microscope was used to observe and save the image.

Protein extraction from *B. subtilis* expression system

The strains stored in the -80 ℃ refrigerator were taken out and streaked on

LB plates containing kanamycin, and cultured at 37 ℃ for 12 h. Single colonies were picked and shaken at 180 rpm for 60 h in LB containing kanamycin. The 15 ml of the culture solution was centrifuged in a 50 ml centrifuge tube at 4 ℃, 10000 rpm for 25 min. The supernatant was filled in a pre-cooled beaker and placed on ice, then, a saturated ammonium sulfate solution was continuously added to the beaker while stirring the liquid in the beaker until the liquid became cloudy. The beaker was placed in a 4 ℃ refrigerator overnight to precipitate the protein, and the fluffy protein was aspirated in a 50 ml centrifuge tube and centrifuged at 4 ℃, 10,000 rpm for 25 min. The supernatant was decanted and the protein was dissolved with 1 ml pre-cooled PBS solution. Each protein concentration was measured by a spectrophotometer, and each protein concentration (including the control protein) was adjusted with a PBS solution, and the adjusted protein was stored in a 4 ℃ refrigerator for use.

Hemolysis analysis

The hemolytic activity was tested with porcine erythrocyte, which was washed three times with PBS (35 mM phosphate buffer, 150 mM NaCl, pH 7.4) and diluted to a final concentration of 1% with PBS. The 75 μl of red blood cell suspension and 75 μl of different concentrations of protein (125, 250, 500, and 1000 ng/μl), 75 μl PBS (negative control) and 75 μl of 0.1% Triton X-100 (positive control), added to 96-well cell culture plates, and incubate at 37 ℃ for 1.5 h. After centrifugation at 4000 rpm for 10 min, 70 μl of the supernatant was taken in a new 96-well cell culture plate, and the results were observed at 450 nm by a microplate reader.

Liquid fermentation and spray drying

The strains stored in the -80 ℃ refrigerator were taken out and streaked on the NA plate containing kanamycin, and cultured at 37 ℃ for 12 h. Single colonies were picked and shaken in 200 ml of kanamycin-containing NA at 170 rpm for 24 h. 1 ml of the culture solution was separately taken in five bottles of 200 ml of kana containing kanamycin at 180 rpm for 48 h (10% inoculum) to finally obtain a *B. subtilis* culture solution. The fermenter was inspected and cleaned, and the prepared NA (5 L) liquid medium was added to the fermenter and sterilized in the fermenter. The fermenter is assembled by Huazhong Agricultural University, College of Life Science and Technology. After the

sterilization is completed, the fermentation speed and temperature are set. When the temperature is lowered to 40 ℃, the alcohol is burned in the sample port, and the *B. subtilis* culture solution is quickly added to the fermenter and fermented for 60 h.

After the fermentation was finished, the fermentation liquid is taken out, the spray drying device is turned on, and the spray drying machine is Triowin's SD-1500. The inlet air temperature and the rotation speed are set so that the obtained powder cannot be too wet or too dry, and the rotation speed is adjusted at any time to stabilize the inlet air temperature. The *B. subtilis* powder is finally obtained.

Preparation of *B. cinerea* conidial suspension

The *B. cinerea* was activated on the PDA, and the activated *B. cinerea* was cultured on a PDA plate for one week. The *B. cinerea* conidia on the PDA plate were washed with 2% glucose and filtered to obtain a conidial suspension. The conidial suspension was adjusted to a concentration of 1×10^6 conidia/ml. The conidial suspension was mixed 1:1 with the diluted *B. subtilis* powder to observe the conidial germination. The picture was taken by microscope NIKON ECLIPSE 55i (NIKON, Japan).

Western blot

Purified protein using Ni-Agarose Resin from CWBIO. The purified protein was isolated by CWBIO's Tricine-SDS-PAGE Gel kit using the pre-stained marker. The isolated protein was transferred to a polyvinylidene difluoride (PVDF) membrane at 21 V and 185 mA for 21 min. The PVDF membrane was shaken with 5% skim milk for 2.5 h at room temperature to block non-specific binding. Next, the PVDF membrane was incubated overnight in 5% skim milk containing His-tag Mouse mAb (1:10000, Frdbio) primary antibody. The PVDF membrane was washed with phosphate-buffered saline containing 0.05% Tween-20 (PBST) and washed three times in 10-15 min. After washing, the PVDF membrane was incubated for 2-4 h at room temperature in 5% skim milk containing Peroxidase AffiniPure Goat Anti-Mouse IgG (H+L) (1:5000, YEASEN) secondary antibody, then the PVDF membrane was washed with PBST (10 min × 3). Enhanced Chemiluminescence (ECL) for protein detection, and analyzed by the image lab.
